# Hunting Increases Phosphorylation of Calcium/Calmodulin-Dependent Protein Kinase Type II in Adult Barn Owls

**DOI:** 10.1155/2015/819257

**Published:** 2015-02-18

**Authors:** Grant S. Nichols, William M. DeBello

**Affiliations:** Department of Neurobiology, Physiology and Behavior, Center for Neuroscience, University of California-Davis, Davis, CA 95618, USA

## Abstract

Juvenile barn owls readily adapt to prismatic spectacles, whereas adult owls living under standard aviary conditions do not. We previously demonstrated that phosphorylation of the cyclic-AMP response element-binding protein (CREB) provides a readout of the instructive signals that guide plasticity in juveniles. Here we investigated phosphorylation of calcium/calmodulin-dependent protein kinase II (pCaMKII) in both juveniles and adults. In contrast to CREB, we found no differences in pCaMKII expression between prism-wearing and control juveniles within the external nucleus of the inferior colliculus (ICX), the major site of plasticity. For prism-wearing adults that hunted live mice and are capable of adaptation, expression of pCaMKII was increased relative to prism-wearing adults that fed passively on dead mice and are not capable of adaptation. This effect did not bear the hallmarks of instructive information: it was not localized to rostral ICX and did not exhibit a patchy distribution reflecting discrete bimodal stimuli. These data are consistent with a role for CaMKII as a permissive rather than an instructive factor. In addition, the paucity of pCaMKII expression in passively fed adults suggests that the permissive default setting is “off” in adults.

## 1. Introduction

Both instructive and permissive signals are needed to guide development and plasticity of neural circuits [1–7]. Instructive signals contain specific information about how a network should be configured to function properly. Permissive signals do not tell a network how to change but must be present for plasticity to occur.

**Figure 1:**
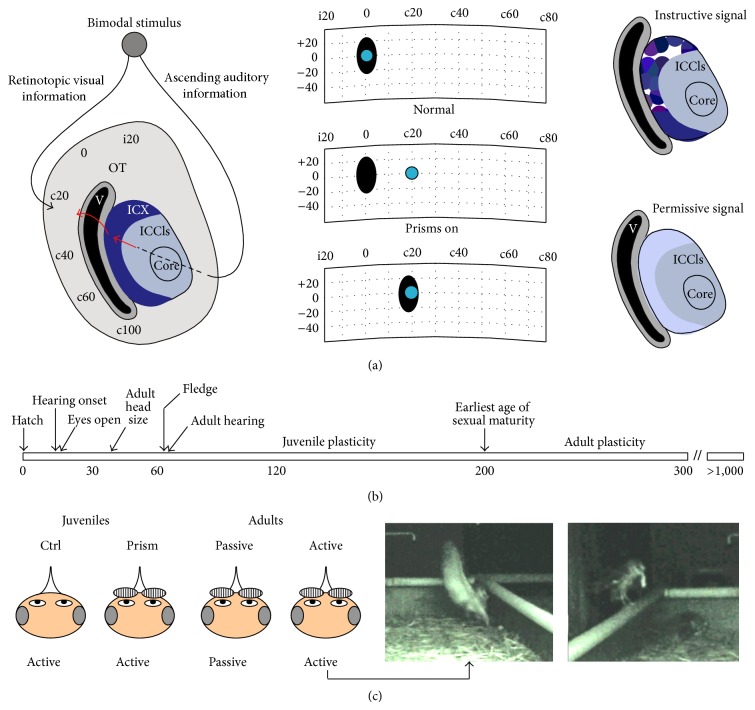
Experimental design. (a)* Left*, diagram of horizontal section through the L midbrain. Auditory information ascends through the core to the lateral shell of the central nucleus of the inferior colliculus (ICCls) where neurons tuned to distinct values of interaural time difference (ITD) are arranged topographically to form a map. ICCls neurons project to the external nucleus of the inferior colliculus (ICX) which contains a complete map of auditory space. Major postsynaptic targets in ICX are CaMKII+ space-specific neurons. In turn, these project to the optic tectum (OT) where the auditory map aligns with a visual map derived from retinotopic input: i20 = ipsilateral 20 degrees; c20 = contralateral 20 degrees. In both ICX and OT, ipsilateral space is represented in the rostral pole and contralateral space progressively towards the caudal pole. V = ventricle.* Middle*, auditory (dark oval) and visual (light circle) spatial receptive fields of a neuron in OT before prism mounting (top), immediately after mounting (middle), and two months later after full adaptation (bottom). The auditory spatial receptive field has reorganized to align with the optically displaced visual field. The primary site of plasticity is the ICX.* Right*, hallmarks of instructive or permissive signals acting at the cellular level within ICX. Instructive signals are expected to produce bimodal effects distributed in patches across rostral ICX, depicted here as multicolor islands. Because prisms do not displace the peripheral visual field, no signal is expected in caudal ICX. In contrast, permissive signals are expected to act across the entire rostrocaudal extent, and with uniform effects at the cellular level, depicted here as constant shading. (b) Developmental timeline of physical features [28, 29] and hearing onset and maturation [30]. Plasticity in juveniles occurs readily with passive feeding on dead mice. After sexual maturity, plasticity in adults requires either active hunting or incremental training. (c) Four experimental groups used in this study: Ctrl (juveniles, no prisms, and active feeding), prism (juveniles, with prisms, and active feeding), passive (adults, with prisms, and passive feeding), and active (adults, with prisms, and active feeding). An example of successful hunting episode recorded with infrared videography is shown on the far right.

The barn owl auditory localization pathway is a useful model for studying the interactions of instructive and permissive signals (Figures 1(a) and 1(b)). Owls, like many animals including humans, localize sounds on the basis of binaural cues [8–10]. These cues are integrated in the external nucleus of the inferior colliculus (ICX) to form a topographic map of auditory space. This map is relayed to the optic tectum (OT; homolog of the mammalian superior colliculus) where it aligns and integrates with a visual space map [11]. Sensory-driven activity in OT triggers movements of the head and neck [12]. Thus, proper map alignment ensures that stimuli originating from a given location in space will orient the animal's gaze and talon strike towards the same location regardless of the modality of the stimulus, which is essential for prey capture [13].

Proper alignment is maintained through the actions of a visually based instructive signal as demonstrated by experimental manipulations that disrupt the spatial register of auditory-visual stimuli, in particular, rearing owls wearing prismatic spectacles [14]. Prisms displace the visual field horizontally, in most studies by 17°–23°. Within ~ two months, the auditory space map in ICX shifts in the direction and magnitude specified by optical displacement, and this circuit-level plasticity restores behavioral integrity. Although the capacity to adapt declines once animals reach sexual maturity [15], plasticity in naïve adults can be achieved by providing the ethologically relevant experience of hunting live mice [16].

The molecular mechanisms underlying plasticity in adult owls are largely unknown. We previously found that activation of cyclic-AMP response element-binding protein (CREB) [17–22] reflects a readout of the instructive information that guides plasticity in juveniles [23]. CREB is activated by several activity-based kinases including the calcium/calmodulin-dependent protein kinase II (CaMKII), a protein known to be crucial for implementing changes in synaptic weight [24–27]. Here we examined CaMKII activation in both juveniles and adults. Using an antibody directed against the active, phosphorylated form of CaMKII (pCaMKII) to label tissue sections, we found evidence for a different role. pCaMKII, unlike pCREB, was not regulated by prism experience in juveniles. However, prism-wearing adults that hunted live mice had elevated levels of pCaMKII within ICX compared to prism-wearing adults that passively fed on dead mice. Also in contrast to pCREB, pCaMKII regulation manifested as a uniform elevation across ICX. We propose that active hunting in adults drives increases in pCaMKII that exceed some critical level necessary for adjustment of the auditory space map. Passive experience may be insufficient to trigger this increase, and insufficient CaMKII activity could account for the failure of these owls to adapt.

## 2. Materials and Methods

*Animals *18 barn owls (*Tyto alba*) were used in this study. All aviary, housing, and experimental procedures were approved by the Institutional Animal Care and Use Committee, University of California, Davis.

### 2.1. Immunoblotting

Brain tissue was isolated from seven normal owls (2 d, 9 d, 14 d, 70 d, 75 d, 300 d, and 2 yrs). Ages of the hatchlings were estimated using a formula based on morphological measurement [28]. Owls were anesthetized with 5% isoflurane in nitrous oxide/oxygen (1 : 1) and perfused transcardially with ice cold sucrose-substituted ACSF. Protein samples were prepared from freshly isolated tectal lobes, homogenized with a Dounce homogenizer in ACSF containing 1 mg/mL trypsin inhibitor, and stored at −20°C. Total protein for each sample was estimated using the Coomassie Plus Protein Assay Kit (Pierce) calibrated with a dilution series of owl forebrain homogenate which was found to be more accurate compared to BSA. For immunoblot analysis, samples were suspended in SDS sample buffer, heated for 4 minutes at 95°C, electrophoretically separated on a 12% SDS-polyacrylamide gel, and transferred to a nitrocellulose membrane (Bio-Rad) overnight. Blots were incubated in Odyssey blocking buffer (Li-COR). Antibodies for immunoblotting were used at the following dilutions (in Odyssey buffer): mouse IgG_1_ anti-*α*CaMKII (Chemicon/Millipore, 6G9 clone; now obtained from GeneTex #GTX41976) 1 : 1000; rabbit anti-phospho-CaMKII (Cell Signaling #3361) 1 : 500; rabbit anti-actin (Sigma #A2066) 1 : 5000; GADPH (ABCAM #AB9485) 1 : 1,000. Secondary antibodies were donkey anti-rabbit 680 (Invitrogen #A10043) or goat anti-mouse 800 (Li-COR #925-32210) at a dilution of 1 : 5000 in PBS containing 0.1% Tween. Imaging was performed on an Odyssey infrared image scanner (LI-COR).

For* in vitro *phosphorylation of CaMKII, samples were diluted 2 : 1 in 60 mM TRIS buffer, pH 7.4 containing 3 mM CaCl_2_ and 60 *μ*g/mL calmodulin (Calbiochem), controls were diluted 2 : 1 in 60 mM TRIS buffer, pH 7.4, and both were incubated at 30°C for 30 minutes.

### 2.2. Surgeries and Prism Mounting

Head bolts and prism mounts were surgically attached to the owls' skull. Anesthesia and postoperative care were provided according to UC Davis Veterinary Care standards. Details of the surgical procedure can be found in [31, 32]. Surgeries were performed at ~65 d of age. After surgical recovery, owls were released into large group aviaries. Subsequently, 19° right-shifting fresnel lenses (prisms) or optically transparent lenses (control) were set in spectacle frames and secured to the prism mount. Prisms were mounted on juveniles at 70–80 d and on adults at >400 d.

### 2.3. Active and Passive Feeding

Active feeding was provided to 5 juveniles and 2 adults. Hunting took place in covered outdoor aviaries just after dusk. The room contained elevated perches that allowed owls to survey the hunting ground, an 8′ × 16′ × 14′′ (width × length × depth) walled zone in the center of the aviary. The floor of the hunting ground was covered with a thin layer of straw. After two days of fasting, prism or control glasses were mounted, the owl was isolated in the room, and two live mice were released. Mice were visible moving along the surface of the straw, and their movements generated localizable sounds. Retrospective videography with an infrared (night vision) attachment confirmed that owls hunted within 10 minutes of the release of the mice (range, 1–10 minutes). To maintain incentive for rapid hunting, owls fasted for two additional days before the final session. Thus, the total duration of prism experience for prism-wearing owls was three days. This duration results in very little, if any, adaptive change in auditory-visual map alignment [33]. Therefore, these animals were experiencing a maximal A-V mismatch.

Passive feeding was provided to 4 adult owls. Thawed dead mice were placed on a small plastic platform with holes drilled in the top to allow transmission of sounds. For 2 owls (augmented passive), a battery-powered music player continually played ethologically insignificant white noise (spectral range 500–6000 Hz) matched in intensity to a mouse moving in straw. The intensity match was determined by recording both the music player and a live mouse moving through straw at the same distance (twelve inches) and then matching the average amplitude. For 2 others (pure passive), no noise was played.

30 minutes after release of the mice into the hunting ground, owls were captured and immediately anesthetized with 5% isoflurane in nitrous oxide/oxygen (1 : 1). Blood was cleared by transcardial perfusion with 0.1 M PB containing 3cc/L bupivacaine, and the animal was perfused with approximately 700 mL of fixative (4% paraformaldehyde in 0.1 M PB) followed by fixative containing 10% sucrose. The brain was removed, postfixed in 4% paraformaldehyde/10% sucrose for 12 hours, and cryoprotected in 30% sucrose/0.1 M PB.

### 2.4. Immunohistochemistry

Immunohistochemistry was performed as previously described [23]. Cryoprotected tissue was cut into 40 *μ*m sections on a freezing microtome. Juvenile tissue and adult tissue were generated in separate experiments and processed separately, but tissue from within these age groups was processed simultaneously using common solutions and processing times to ensure consistency. Sections were blocked at room temperature for one hour in 0.1 M phosphate buffer (PB) containing 4% normal goat serum (Vector Laboratories, Burlingame, CA), 1% bovine serum albumin (Fisher, Hampton, NH), and 0.4% Triton X-100. Primary antibodies were diluted in 0.1 M PB, 1% normal goat serum, 1% bovine serum albumin, and 0.3% Triton X-100 as follows: mouse IgG_1_ anti-*α*CaMKII (1 : 500); rabbit polyclonal anti-pCaMKII (1 : 200); anti-EF1*α* (Chemicon, 1 : 1,000); mouse anti-DARPP-32 (gift of HC Hemmings, Weill Cornell Medical College, 1 : 5,000); tyrosine hydroxylase (Chemicon, 1 : 1,000), and incubated overnight at 4°C. Sections were rinsed 2 × 5′ in 0.1 M PB at RT and incubated for one hour with secondary antibodies diluted 1 : 1000 in 0.1 M PB containing 0.02% Triton and 0.25% BSA. Secondary antibodies were goat anti-mouse 488 and goat anti-rabbit 568 from Molecular Probes (Invitrogen, Carlsbad, CA). After incubation with secondary antibodies, sections were rinsed in 0.1 M PB containing 0.25% BSA and subsequently in 0.1 M PB, mounted on coverslips in dH20 containing 0.3% gelatin, allowed to dry, mounted on slides with Vectashield Hardset Mounting medium (Vector Laboratories), and sealed with nail polish. One set of sections not used for quantitative analysis was processed with a very low concentration of anti-*α*CaMKII (1 : 100,000) and visualized using tyramide signal amplification (TSA; Molecular Probes) according to the manufacturer's instructions.

### 2.5. Microscopy and Image Analysis

Imaging was performed on a Zeiss LSM 510 confocal microscope using a 63x oil-immersion lens, numerical aperture 1.4. Confocal image stacks were acquired from the rostal and caudal ICX in tissue sections from middorsoventral levels of the tectal lobe [34]. Images were acquired from the ICX only, based on the gradient of *α*CaMKII staining [35]. Image location in rostral or caudal ICX was assigned based on previously described criteria [23].

The AF 488 secondary antibody was excited using a 488 nm laser line and collected using a 500–530 nm bandpass filter. The AF 568 secondary antibody was excited using a HeNe 543 nm laser line and collected using a 565–614 nm bandpass filter. The fluorophores were excited sequentially rather than simultaneously, and no cross-channel bleed-through was observed using these settings. For most sections and color channels, laser intensity and detector gain were adjusted to utilize full dynamic range while minimizing the number of saturated pixels, typically resulting in pixel intensity values that ranged from 10 to ~200 across the image field. Differences in acquisition settings for each channel were corrected for as previously described [23] and staining for each measured region was calculated by subtracting the acquisition gain-corrected background staining from the corrected measured region. For the *α*CaMKII channel in juveniles only, the range acquired was compressed by ~10-fold. The only impact of this on the analysis is that the range of pCaMKII/CaMKII values reported in Figure 4 is larger in juveniles than adults. This should have no impact on interpretation because the comparisons for this analysis are within, not across, age groups, and the detector response is linear over this range.

Confocal image stacks were analyzed in ImageJ. Every distinct perikaryl profile stained with either *α*CaMKII or pCaMKII staining was analyzed. The perikarya in the optical section with the greatest cross-sectional area was manually traced on the basis of the more intense of the two color channels. The border of the nucleus was traced as the abrupt drop-off from perinuclear staining. This yielded two ROIs: the* perinuclear region* localized to the cytoplasm of the neuronal cell body, in which staining was typically uniform, and the* nuclear region*, in which staining typically occurred within puncta. Mean signal intensities (0–255) of each color channel in each ROI are reported.

Data analysis and statistics were performed in IgorPro (Wavemetrics). Most distributions shown in Figures 4, 5(b), 5(c), and 6 were not normally distributed; thus, *P* values are reported from the Mann-Whitney *U* test. Significance criterion was *P* < 0.01. Data for the pure passive and augmented passive groups were not significantly different (*P* = 0.628, Mann-Whitney *U* test) and therefore all passively fed adults were pooled into one experimental group for analysis. Individual data from three of four passive adults was significantly different from the pooled active hunters (*P* < 0.01, Mann-Whitney *U* test). The data in Figure 4 was case normalized within experimental group by randomly culling cells from oversampled individuals. The data in Figure 5 was not case normalized because the comparisons are within individuals. Thus, the overall mean values reported in these two figures are slightly different.

## 3. Results

The main goal was to investigate phosphorylation of CaMKII and CREB within the ICX of prism-adapting juvenile and adult owls. First, we analyzed the developmental regulation of CaMKII and CREB expression in brain homogenates derived from normal owls from hatchling to adult. Second, we analyzed experience-dependent regulation of pCaMKII at the level of individual neurons using immunohistochemistry. This analysis applied to the four experimental groups described in Figure 1(c) and comprises the bulk of the data. Finally, we report that CREB detection failed conspicuously in adult tissue despite the general competence of adult tissue for immunohistochemistry. Nonetheless, the results for pCaMKII regulation stand in contrast to previously published results for CREB regulation in juveniles.

### 3.1. Developmental Regulation of CaMKII and CREB Expression

**Figure 2:**
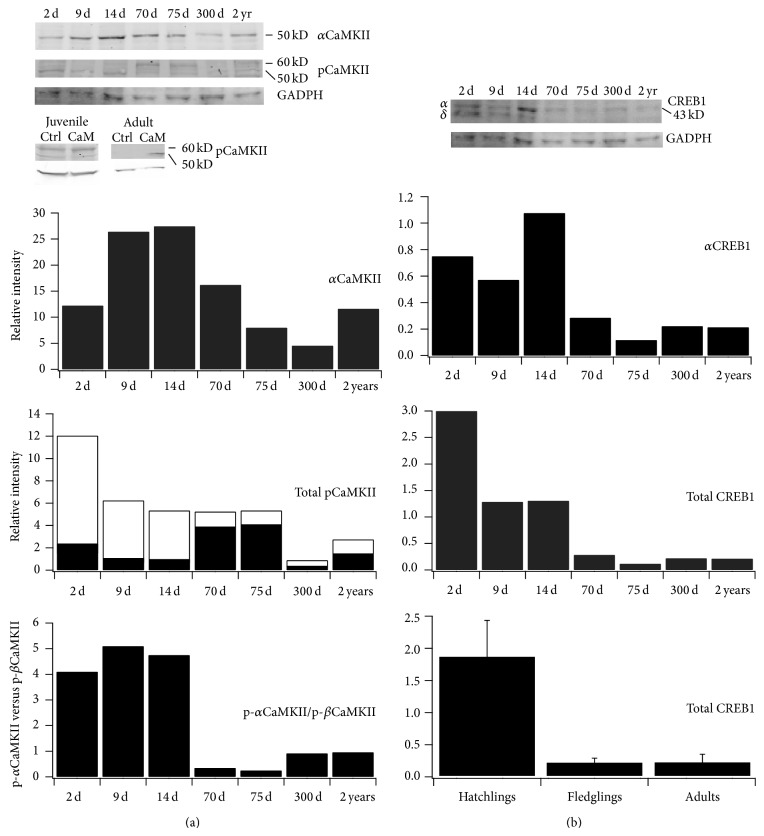
Immunoblotting. Brain homogenates of 7 owls ranging in age from 9 d to 2 yrs were analyzed using antibodies against native (*α*CaMKII) and phosphorylated (pCaMKII) forms of CaMKII and native CREB1. (a)* Top*, *α*CaMKII labeled a single band of expected molecular weight, 50 kD. pCaMKII labeled two distinct bands, phospho-*α*CaMKII (50 kD) and phospho-*β*CaMKII (60 kD). GADPH was used as loading control. Protein samples from a single juvenile and single adult in the absence (ctrl) and presence (CaM) of calmodulin confirm the ability of the phosphospecific antibody to detect changes in phosphorylation state.* Bottom*, normalized intensities for *α*CaMKII, pCaMKII, and their ratio. For total pCaMKII, black bars correspond to phospho-*β*CaMKII and open bars to phospho-*α*CaMKII. (b)* Top*, CREB1 labeled a single band (in most samples) of expected molecular weight, 43 kD, corresponding to the alpha isoform of CREB1. Hatchlings also expressed lower levels of the delta isoform.* Bottom*, normalized intensities for *α*CREB1, total CREB1, and total CREB1 binned for hatchlings (2, 9, and 14 d), fledglings (70 and 75 d), and adults (300 d and 2 yr).

Developmental regulation of CaMKII and CREB, and their phosphorylated isoforms, was characterized using immunoblots (Figure 2). Protein samples were from three hatchlings prior to the age of eye opening (2, 9, and 14 d), two fledglings (70 and 75 d) that had just learned flight, and two adults. None of these owls had a history of prism experience.

Consistent with previous reports, *α*CaMKII antibody (6G9 clone formerly obtained from Chemicon and Millipore, currently obtained from GeneTex) labeled a single band of the expected molecular weight, 50 kD. Expression peaked in the 14 d sample and then dropped but was similar between fledgling and adult, which subsumes the age range used in the prism/behavior experiments. The phosphospecific antibody (pCaMKII) recognized a 50 kD band corresponding to phosphorylated-*α*CaMKII and another band at 60 kD, corresponding to the phosphorylated *β* isoform of CaMKII. This isoform is expressed at low levels in hatchlings and adults, with strong expression in fledglings. In hatchlings, phosphorylated-*α*CaMKII predominated. In fledglings, this ratio reversed. In adults, the relative amounts of phosphorylated-*α*CaMKII and phosphorylated-*β*CaMKII were similar.

*In vitro* phosphorylation assays confirmed the ability of pCaMKII antibody to track changes in phosphorylation state (Figure 2). Incubation of homogenate from a 70 d juvenile with Ca^2+^ and calmodulin resulted in more intense staining (~30%) of the 60 kD phosphorylated-*β*CaMKII band and no apparent regulation of the 50 kD phosphorylated-*α*CaMKII band. In a dilute sample from an adult that exhibited no detectable pCaMKII prior to incubation with Ca^2+^ and calmodulin, the 50 kD band was readily apparent following treatment, while 60 kD band was not.

Consistent with previous reports, CREB antibody labeled a single band of expected molecular weight, 43 kD. Expression was similar in the three hatchlings and lower in fledglings and adults (*P* = 0.018, Student's *t*-test). An additional band corresponding to delta CREB [36] was present in hatchlings but not detectable in fledglings and adults.

### 3.2. Expression of *α*CaMKII and pCaMKII in ICX

**Figure 3:**
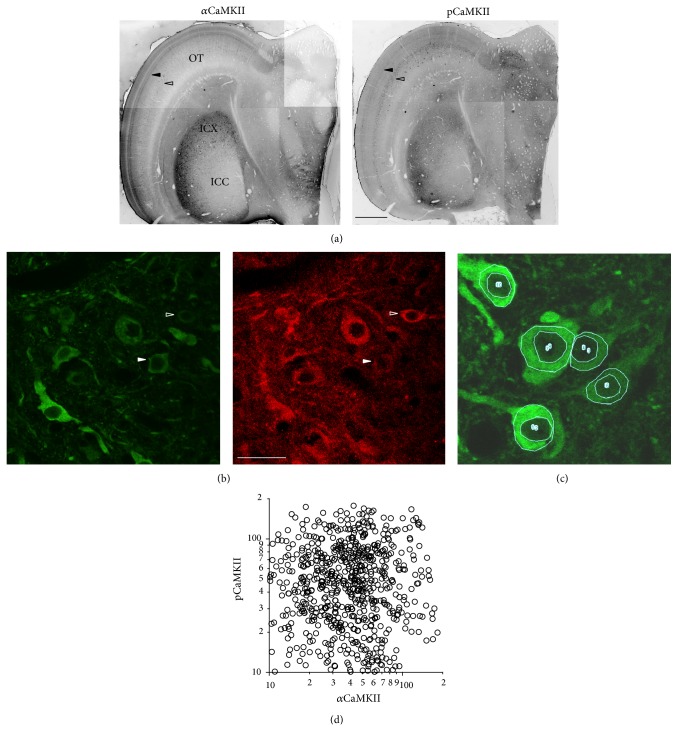
Immunohistochemistry. (a) Regional distribution of *α*CaMKII (left) and pCaMKII (right). Montages were constructed from low magnification images of a single horizontal section (40 *μ*m) through the L midbrain of an adult owl. The red and green color channels were converted to grayscale for viewing only. Within the inferior colliculus, both proteins were most strongly expressed in ICX, the site of plasticity. Within the optic tectum, *α*CaMKII was most strongly expressed in layers 8 and 10 (solid arrowhead), whereas pCaMKII was most strongly expressed in layers 12/13 (hollow arrowhead). (b) Subcellular distribution of *α*CaMKII (left) and pCaMKII (right) in ICX. Shown is a single optical section from a high magnification confocal stack. Both proteins were localized to the same population of cells and both expressed in the perikarya. The relative intensity of staining varied from cell to cell (compare open and hollow arrowheads). (c) Perinuclear ROIs were manually traced for every neuron in the image field (*n* = 5 neurons in this example); nuclear staining mostly occurred in small punctae (one or two per nucleus in this example) and was measured separately. (d) Signal intensities for each perinuclear ROI in adult owls (6 owls, 804 cells). There was no correlation between *α*CaMKII and pCaMKII signals (*R* = −0.014, *P* = 0.78).

The regional distributions of *α*CaMKII and pCaMKII immunolabeling in the tectal lobe are shown in Figure 3(a). Within the inferior colliculus, pCaMKII labeling tracked closely with *α*CaMKII labeling, with both being most strongly expressed in ICX, the major site of plasticity during prism adaptation. Labeling was lower in the ICCls and essentially absent from the ICCcore, and results are consistent with previous reports for *α*CaMKII labeling in owl [35] and chicken [37, 38]. In comparison, a differential labeling pattern was observed in OT, with *α*CaMKII most strongly expressed in the superficial/intermediate layers and pCaMKII most strongly expressed in the deeper layers (arrowheads in Figure 3(a)).

Subcellular localization was revealed using high magnification confocal imaging (Figure 3(b)). pCaMKII was abundant in perikaryal and proximal dendritic regions, highly expressed in synaptic punctae, and sparingly expressed (above background) in nuclei. Almost all neurons that expressed one expressed the other, although the relative intensities vary widely from neuron to neuron; for some cells, pCaMKII intensity was low while *α*CaMKII was high (solid arrowhead, Figure 3(b)), and for others, pCaMKII was high and *α*CaMKII was low (empty arrowhead, Figure 3(b)). Signal intensities for both antibodies were measured within the perikaryal region of every neuron in each image field (Figure 3(c)). Scatter plot of measurements from all neurons in adult owls is shown in Figure 3(c). There was no correlation between *α*CaMKII and pCaMKII signal intensity. Cells in which pCaMKII > *α*CaMKII may reflect the fact that the *α*CaMKII antibody recognizes only one isoform of CaMKII, while the pCaMKII antibody recognizes all isoforms, including the *β* isoform, which, along with *α*CaMKII, constitutes the bulk of CaMKII in the brain [39]. Overall, these data indicate that perikaryl pCaMKII is differentially activated on a cell-by-cell basis. Synaptic punctae were not quantified for reasons presented in the Discussion.

### 3.3. pCaMKII in ICX Is Regulated by Hunting, Not Prism Experience

**Figure 4:**
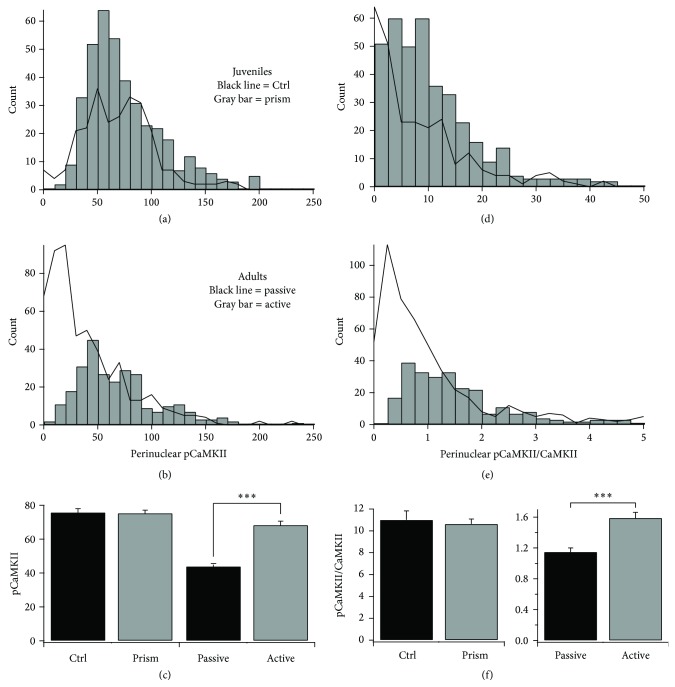
Experience-dependent regulation of perinuclear pCaMKII.* Left panels*, frequency histograms of perinuclear pCaMKII signal in (a) Ctrl versus prism juveniles and (b) passive versus active adults. (c) Mean values for all four experimental groups. No difference was observed between controls and prism-wearing juveniles (Mann-Whitney *U* test, *P* = 0.29). In contrast, active hunting in adults significantly increased perinuclear pCaMKII intensities (^***^ *P* < 0.0001 for Mann-Whitney *U* test).* Right panels*, frequency histograms of perinuclear pCaMKII/CaMKII signals in (d) Ctrl versus prism and (e) passive versus active. (f) Mean values for all four experimental groups. A small magnitude difference was observed between Ctrl and prism juveniles but was not significant (Mann-Whitney *U* test, *P* = 0.02). In contrast, active hunting in adults significantly increased perinuclear pCaMKII/CaMKII signals (^***^ *P* < 0.0001 for Mann-Whitney *U* test). All data in this figure is case normalized within experimental group: control juveniles (262 cells), prism-wearing juveniles (392 cells), passive adults (525 cells), and active adults (270 cells).

To investigate whether CaMKII is regulated by ethologically relevant experience we conducted behavioral experiments with four groups of owls (Figure 1(c)). In juveniles that actively hunted live mice in the 30 minutes prior to sacrifice, no differences were observed in pCaMKII regulation between control and prism-wearing owls (Figures 4(a) and 4(c)). In contrast, in prism-wearing adults, pCaMKII was on average elevated in active hunters versus passive feeders (Figures 4(b) and 4(c)). The same pattern of results and statistical significance was observed when the pCaMKII signals were normalized for CaMKII signal intensity (Figures 4(d), 4(e), and 4(f)). Thus, hunting in adults, not prism experience in juveniles, activated CaMKII.

To determine whether hunting provided an instructive signal mediated by CaMKII we examined three aspects of the cell-specific pCaMKII distributions [23] (Figure 1(a)): (1) shape change (lateral shift versus bimodal), (2) localization to rostral versus caudal ICX, and (3) “patchiness,” that is, deviation in the mean intensity values from one image field to the next, compared with relevant control group.

**Figure 5:**
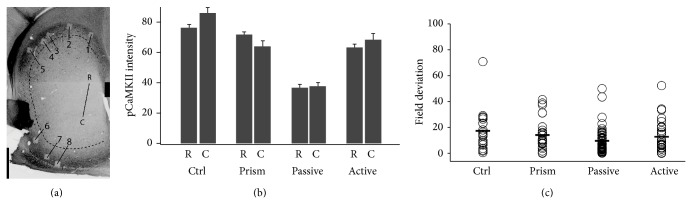
pCaMKII regulation is uniform across the ICX. (a) Representative section after high magnification imaging. Photobleaching of the 63x confocal image fields confirms their location within ICX. Fields 1–5 are located in rostral ICX, which represents frontal auditory space and is subject to optical displacement. Fields 6–8 are located in caudal ICX, which represents peripheral auditory space and is not subject to optical displacement. Rostrocaudal location was assessed according to the criterion in Nichols and DeBello, 2008. (b) Mean pCaMKII intensities for R (rostral) and C (caudal) image fields in all four experimental groups. The differences are small, inconsistent across groups, and not significant (minimum *P* value, Ctrl R versus C, *P* = 0.034). (c) Field deviation is the percentage change in mean signal intensity from one image field to its nearest neighbor. Neither comparison was significant: Ctrl versus prism, *P* = 0.53; passive versus active, *P* = 0.39.

In this study, none of these hallmarks were observed. In passive versus active adults, the change in the pCaMKII intensity distribution was a lateral shift with no indication of bimodality (Figures 4(b) and 4(e)); no rostrocaudal differences were apparent (Figure 5(b)), and field deviations were small and indistinguishable (Figure 5(c)). In control versus prism-wearing juveniles, there was no change in pCaMKII intensity distribution; small rostrocaudal differences were apparent yet trended in the opposite direction for each experimental group and thus were not consistent (Figure 5(b)), and field deviations were small and indistinguishable (Figure 5(c)). In total, these data do not support an instructive role for pCaMKII.

**Figure 6:**
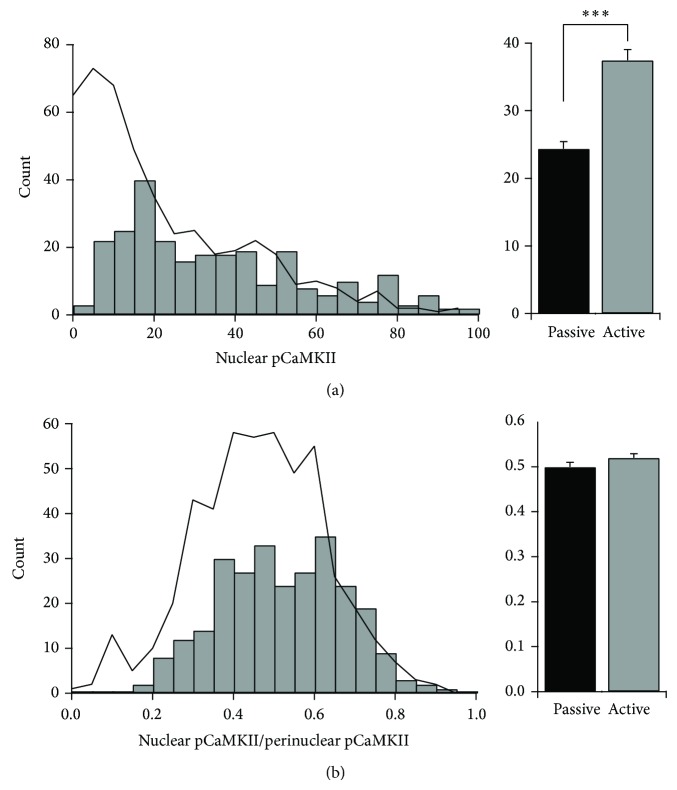
Experience-dependent regulation of nuclear pCaMKII. (a)* Left*, frequency histograms of nuclear pCaMKII signal in passive (black line) versus active adults (gray bars).* Right*, mean values significantly increased (^***^ *P* < 0.0001, Mann-Whitney *U* test). (b)* Left*, frequency histograms of nuclear pCaMKII/perinuclear pCaMKII in passive (black line) versus active adults (gray bars).* Right*, mean values were similar in magnitude and not significantly different (*P* = 0.02, Mann-Whitney *U* test).

pCaMKII was also observed in nuclei. This signal was elevated by active hunting (Figure 6(a)) and in direct proportion to the elevation in perinuclear pCaMKII (Figure 6(b)).

### 3.4. CREB Immunohistochemistry in Adult Tissue

**Figure 7:**
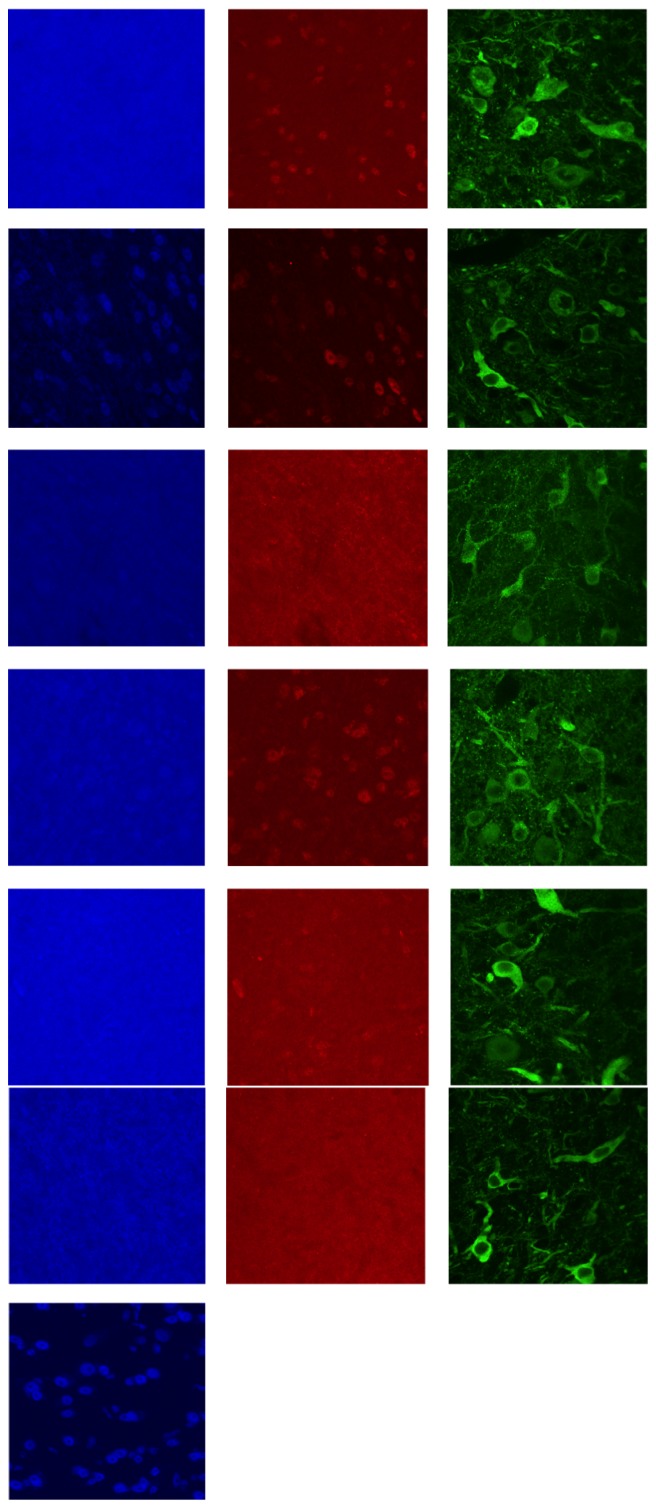
Failure of CREB immunohistochemistry in adult tissue. High magnification images in ICX from sections stained with CREB (blue), phospho-CREB (red), and CaMKII (green). Each horizontal row corresponds to a different adult owl (6 total). Bottom left panel is tissue from a juvenile owl that reacted with the same lot of anti-CREB antibody.

The pattern of CREB regulation in juvenile (fledgling) owls promised a tool to monitor the delivery of instructive information to ICX, with potential to analyze the proximate causes of plasticity failure in adults (see Discussion). We twice attempted to apply this tool to separate cohorts of adults, those reported in Figure 7 and an additional five owls from whom data is not shown; in both cases, immunohistochemical detection of CREB and pCREB failed almost completely.

**Figure 8:**
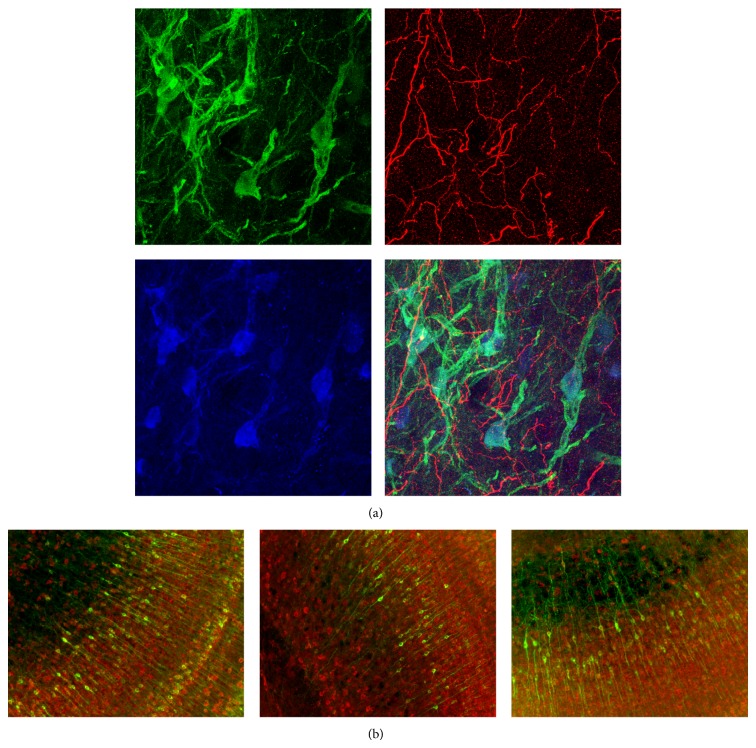
General success of immunohistochemistry in adult tissue. (a) Immunostaining for CaMKII (green), tyrosine hydroxylase (red), DARPP-32 (blue), and overlay (bottom right). (b) Ultrasensitive detection of *α*CaMKII with primary antibody dilution at 1 : 5,000 (left), 1 : 20,000 (middle), and 1 : 100,000 (right). Red channel is EF1*α* at 1 : 1,000 detected with standard procedure.

Three potential explanations were examined: (1) developmental downregulation of CREB from fledgling to adult: the immunoblotting data in Figure 2 make this unlikely, (2) changes in the lot-to-lot efficacy of the commercial antibodies: this also is unlikely, because immunostaining of juvenile tissue with the same aliquot of CREB antibody that failed in adults was excellent (Figure 7, bottom left panel) and equivalent in quality to previously published results with juvenile tissue, and (3) age-related decrease in antibody access to paraformaldehyde-fixed intracellular signaling proteins: yet, immunostaining for CaMKII, tyrosine hydroxylase, and DARPP-32 was excellent in adult tissue (Figure 8(a)), with CaMKII detectable at primary antibody concentrations down to 1 : 100,000 (Figure 8(b)). Thus, it appears that the age-related decline in immunostaining quality was, among these four proteins, specific to CREB.

## 4. Discussion

The capacity for prism adaptation is delimited by age and experience [15]. Juveniles who receive prisms at the age of fledging (~60 d) are capable of adapting to large-scale optical displacements (>17°) even in impoverished conditions, whereas juveniles who receive prisms at later ages, but before sexual maturity (~200 d), require standard conditions of free flight and passive feeding on dead mice. When naïve adults are challenged with the same prisms and conditions, they do not adapt [15]. This plasticity failure could result from failure to deliver instructive information to ICX or failure of ICX to respond to that information. We set out to resolve this issue using pCREB/CREB activation as a tool to monitor delivery of instructive signals [23]. Unfortunately, detection of CREB was poor in adults (Figure 7). A similar anomaly was observed for the postsynaptic protein Homer1 (McBride and DeBello, manuscript in review), despite the general competence of adult tissue for immunohistochemistry (Figure 8). Protein complexing and/or maturation of extracellular matrix could impose a barrier for certain antibodies, and if so, CREB analysis could be revisited using antigen unmasking [40], light fixation [41], or ultrathin sections and array tomography [42, 43].

In contrast, immunodetection of pCaMKII and CaMKII was quite good in adults (Figure 3) and permitted investigation of CaMKII activation. Consistent with previous reports across species [44], pCaMKII was observed in the cytoplasmic compartment surrounding neuronal nuclei (to a much lesser extent within nuclei) and in synapse-sized punctae and short segments of axons and dendrites rife throughout ICX. Both of these subcellular compartments are of interest; however, interpretation of perikaryal pCaMKII is more straightforward than for synaptic/neuropil pCaMKII. First, perikarya correspond to space-specific neurons (SSNs) that exhibit sharp tuning for auditory cues. In contrast, the synaptic/neuropil punctae represent synapses encoding a wide range of auditory cues, because the axonal projections that give rise to these synapses are broad [32, 45]. Since neighboring punctae are functionally heterogeneous, the value (+ or −) of the instructive signal received by each synapse cannot be known in the absence of tracking axons back to their source locations; thus, pCaMKII values for these punctae would be uninterpretable. Second, it is likely that many punctae do not represent synapses but transport packets of pCaMKII. Costaining with other synaptic markers could be used to determine the fraction actually localized to synapses, but as noted above, immunodetection of synaptic proteins in adult tissue can be sporadic and yield results contaminated with high false negatives. Thus, we focused exclusively on quantifying perikaryal pCaMKII.

We first compared control and prism-adapting juveniles, with both groups actively hunting. The prism owls had only three days of prism experience, and because no behavioral or functional changes in auditory localization take place within this frame, these individuals were experiencing a 19° mismatch, large instructive signals. Yet the distributions of pCaMKII/CaMKII values were indistinguishable (Figure 4). We conclude that activation of perikaryal CaMKII neither encodes the instructive signal nor represents a permissive signal activated by A-V mismatches.

We next compared prism-wearing adults fed passively on dead mice to those that actively hunted. In passive adults, pCaMKII/CaMKII values were lower than in juveniles but were elevated in active hunters (Figure 4). However, this CaMKII activation did not bear the hallmarks of instructive information (Figure 5). We propose that pCaMKII represents a permissive signal turned “on” by the engaging task of hunting. Because the absolute level of pCaMKII was low in passive adults who do not adapt to prisms, this default setting for this permission past the age of sexual maturity appears to be “off.” Because juveniles adapt with or without hunting, we predict the default setting in the juvenile brain to be “on.” This prediction could be tested by measuring pCaMKII expression in passive versus active juveniles.

What turns on permissive pCaMKII in adult hunters? One possibility is allocation of top-down attention mediated by the arcopallial gaze fields (AGF), homolog of the mammalian frontal eye fields [46]. The AGF is one of only three brain structures known to project to ICX, and microstimulation of AGF effects neural gain [47] in the midbrain. If AGF is required to turn on permissive pCaMKII, lesions of the AGF should prevent hunting-dependent plasticity in adults but not prism adaptation in juveniles.

A nonexclusive possibility is arousal mediated by modulatory pathways such as the cholinergic nucleus basalis, dopaminergic nucleus accumbens, and/or noradrenergic locus ceruleus. Direct projections from these structures to the ICX have not been reported in owls; however, polysynaptic pathways exist via the OT, the cholinergic nucleus isthmi pars parvocellularis [48], and/or the inhibitory isthmi pars magnocellularis [49]. Activation of nucleus basalis is required for auditory cortical plasticity in adult rats but not rat pups [50, 51], evocative of the requirement for active hunting in adult owls.

How might activation of pCaMKII enable adult plasticity? We focused on perikaryal pCaMKII. From visualization of 3D confocal image stacks, a large proportion of this signal was in the cytoplasm. Previous work has focused primarily on CaMKII translocation to synaptic membranes and its role in promoting long-term potentiation by coupling synaptic activation to changes in postsynaptic AMPA receptor content and NMDA receptor conduction [25, 52, 53]. Less is known regarding the role of cytoplasmic CaMKII. It has been shown that target selection of pCaMKII depends on membrane versus cytoplasmic localization, which raises the possibility that cytoplasmic pCaMKII phosphorylates different targets, those directly involved in LTP. A model suggested by our data is that cytoplasmic pCaMKII is required (permissive) to implement the instructive signals encoded by pCREB. This could occur by convergence of these pathways in the nucleus (the *α*_*B*_ CaMKII isoform is known to translocate to the nucleus) or because pCaMKII is required to modify new proteins produced by CREB activation. These proteins would be distributed as cell-wide resources and interact with synaptic tags containing synapse-specific instructive information. We cannot rule out the possibility that synaptically localized CaMKII—not analyzed here for reasons stated earlier—may contribute to the synaptic tag and therefore also serve an instructive role.

A comprehensive survey of studies on the consolidation phase of avoidance conditioning in rats and chicks concluded that, in at least some paradigms, PKC and PKA provided permissive signals for memory formation, whereas CaMKII played an instructive role [54]. This was based on behavioral assays and systemic pharmacological manipulations. The main line of evidence was a* lack* of net activation of CaMKII during consolidation despite a strong effect of CaMKII inhibitors on consolidation. Consistent with the interpretations presented here, this is expected from a distributed memory model where some synapses strengthen and others weaken.

A recent study examined CaMKII activation in rats following unsupervised learning [55]. Two groups were analyzed 30 minutes after exploring a complex environment with (contingency) or without (unsupervised) an aversive cue that limited the scope of exploration. Immunohistochemical detection was used to create maps of pCaMKII+ synapses in 21 sampling zones across several structures in the hippocampus. Correlations between groups were strong across most zones, yet the number of PCaMKII+ synapses was elevated on average in the medial CA1a stratum oriens, medial CA3 stratum lacunosum-moleculare, and medial CA3c stratum oriens. These results are consistent with an instructive role for CaMKII at individual synapses within these substructures.

Neither of the above studies analyzed perikaryal CaMKII. Taken together, these and our data suggest a model in which cytoplasmic CaMKII activation plays a permissive role in long-term plasticity while CaMKII activation in synaptic membranes and PSD plays an instructive role. These roles would be achieved by compartment-specific targeting of substrates that interact with either cell-wide resources (permissive) or synaptic tag proteins (instructive). A complete list of candidate substrates is not yet known [53].
